# A mixed methods study on medicines information needs and challenges in New Zealand general practice

**DOI:** 10.1186/s12875-021-01451-7

**Published:** 2021-07-10

**Authors:** Chloë Campbell, Rhiannon Braund, Caroline Morris

**Affiliations:** 1 School of Pharmacy, University of Otago, Dunedin, New Zealand; 2 Department of Primary Health Care and General Practice, University of Otago, Wellington, New Zealand; 3 Pharmaceutical Society of New Zealand, Wellington, New Zealand; 4 New Zealand Pharmacovigilance Centre, University of Otago, Dunedin, New Zealand

**Keywords:** Family practice, General practice, Primary health care, Drug prescribing, Information storage and retrieval, Medicines information

## Abstract

**Background:**

Medicines are central to healthcare in aging populations with chronic multi-morbidity. Their safe and effective use relies on a large and constantly increasing knowledge base. Despite the current era of unprecedented access to information, there is evidence that unmet information needs remain an issue in clinical practice. Unmet medicines information needs may contribute to sub-optimal use of medicines and patient harm. Little is known about medicines information needs in the primary care setting. The aim of this study was to investigate the nature of medicines information needs in routine general practice and understand the challenges and influences on the information-seeking behaviour of general practitioners.

**Methods:**

A mixed methods study involving 18 New Zealand general practitioner participants was undertaken. Quantitative data were collected to characterize the medicines information needs arising during 642 consultations conducted by the participants. Qualitative data regarding participant views on their medicines information needs, resources used, challenges to meeting the needs and potential solutions were collected by semi-structured interview. Integration occurred by comparison of results from each method.

**Results:**

Of 642 consultations, 11% (n = 73/642) featured at least one medicines information need. The needs spanned 14 different categories with dosing the most frequent (26%) followed by side effects (15%) and drug interactions (14%). Two main themes describing the nature of general practitioners’ medicines information needs were identified from the qualitative data: a ‘common core’ related to medicine dose, side effects and interactions and a ‘perplexing periphery’. Challenges in the perplexing periphery were the variation in information needs, complexity, ‘known unknowns’ and ‘unknown unknowns’. Key factors affecting general practitioners’ strategies for meeting medicines information needs were trust in a resource, presence of the patient, how the information was presented, scarcity of time, awareness of the existence of a resource, and its accessibility.

**Conclusions:**

General practitioners face challenges in meeting wide-ranging medicines information needs in patients with increasingly complex care needs. Recognising the challenges and factors that influence resource use in practice can inform optimisation of medicines information support resources. Resources for general practitioners must take into account the complexity and time constraints of real-world practice. An individually responsive approach involving greater collaboration with pharmacists and specialist medicines information support services may provide a potential solution.

**Supplementary Information:**

The online version contains supplementary material available at 10.1186/s12875-021-01451-7.

## Background

The optimal use of medicines is a fundamental goal in health policy worldwide, especially given the growing pressure from chronic multi-morbidity in aging populations increasing the potential for inappropriate polypharmacy and negative ramifications for patient outcomes [1, 2]. 

The safe and effective use of medicines relies on a large, constantly increasing knowledge base [3]. Despite the current era of unprecedented access to information, there is evidence that unmet information needs remain an issue in clinical practice [4]. Unmet needs are not simply due to a lack of information; rather, the new challenge is ‘information overload’ [5]. Consequences of information overload include failure to process all available information, incorrect processing of information, accepting lower quality information, and abandoning the search for needed information [6]. Information failures may contribute to less than optimal use of medicines or medicines-related harm [7].

It is recognised that doctors regularly have information needs or questions relating to the care of their patients and that keeping up to date is a constant challenge [8]. Research characterising information needs in primary care suggests that questions about medicines dominate [9–11].

The main barriers identified in the literature preventing primary care physicians from meeting their general clinical information needs are lack of time and skills to complete a search and appraise information efficiently and effectively [5, 12]. However, literature specifically addressing the challenges and barriers to meeting *medicines* information needs in general practice is lacking. Research that focuses on the *medicines* information-seeking behaviour of general practitioners^1^ (GPs) is very limited and dated, though pharmacists and medicine compendiums have been identified as useful to assist in appropriate prescribing [6]. Given health system emphasis on medicines optimisation in the face of chronic multi-morbidity and ongoing rapid evolution of the information environment, a clearer understanding of the present situation is important. The aim of this study was to identify the medicines information needs of GPs during routine clinical practice, the strategies they use to meet these needs, and the challenges they face. Improved understanding in these areas will help to inform enhancement of medicines information resources and support services for primary care^2^ and therefore contribute positively to the optimal use of medicines as well as harm reduction.

## Methods

A mixed methods approach with a convergent design [13] was used to investigate GPs’ medicines information needs. This approach allowed the selection of methods best suited to answering the research questions. The two methods used were:

1. Quantitative descriptive research using a ***structured reflection*** template to capture data about the medicines information needs arising and the information-seeking strategies used by GPs over two clinic days.
2. Qualitative interpretive research using a follow-up face-to-face ***semi-structured interview*** to explore GP views on their medicines information needs including resources used, challenges experienced in practice and potential solutions.

Following approval by the University of Otago Human Ethics Committee (reference number D15/314) a purposive sampling strategy was used, recruiting GPs in active clinical practice from two areas of New Zealand: a metropolitan city and a smaller provincial city, and their surrounds. Flyers advertising the study were sent to the practice manager of all general practices in the provincial city (n = 28) and an equivalent number in the metropolitan city. Practice managers distributed the flyers to GPs within their practice. GPs contacted the research team if they were interested in participating. Informed consent was obtained from all participants. They were offered a voucher as a token of appreciation for their willingness to participate. This incentive was considered important to attain sufficient participant numbers recognising the time constraints of GPs and that they are a highly researched group [14].

For the structured reflection, one of the authors (CC) met face-to face with each GP individually. During this meeting, GPs used the appointment list within their patient management system to verbally reflect on each consultation during the two preceding clinic days. They were prompted to describe the medicines information needs arising and resources used by questions from the researcher (see Additional file 1 for data collection template). To reduce the potential for memory recall bias, participants had also been provided with a documentation booklet (see Additional file 2) in advance of the two clinic days so they could make notes about the medicines information needs arising and resources used. During the structured reflection, key information was documented by the researcher using the data collection template. The meeting was also digitally recorded with participant consent and the audio recording used to verify and supplement data captured by the researcher. The structured reflection generated quantitative, descriptive data about the nature of medicines information needs arising and the resources used to meet them. This data was managed in Microsoft Excel and processed using descriptive statistics.

Structured reflection is an adapted version of post-consultation interview, a method used in previous research examining the information needs of clinicians [6]. This approach enabled quantitative data about the medicines information questions arising in general practice to be gathered without the disruption to participants of being questioned after each individual consultation. It also reduced the potential for memory bias compared to survey [15].

The semi-structured interviews were undertaken (also by CC) after the structured reflection, usually with an interval of 1–2 weeks. Some were undertaken immediately afterwards if that was more convenient for the participant. They were guided by an interview schedule (see Additional file 3) which focused on the nature of their medicines-related information needs, the resources they prefer to use, challenges experienced in practice, and potential solutions. These interviews were digitally recorded with participant consent, and transcribed verbatim. As an accuracy check, participants were given the opportunity to review their transcript and make amendments if necessary. The qualitative data were managed in NVivo (version 10, QSR International Pty Ltd) and an inductive thematic analysis undertaken [16]. This involved iterative reading of transcripts and coding for potential themes and subthemes by CC. A second member of the research team (CM) read and independently coded six randomly selected transcripts. No new themes emerged during the latter interviews indicating that data saturation had been attained. The final thematic framework was discussed and agreed by all three authors. The analysis process considered both recurrent and outlying concepts emerging from the data. All members of the research team are pharmacists and have a professional interest in medicines information needs arising in healthcare and consider that unmet needs could hinder optimal outcomes from the use of medicines. Reflexivity was practised throughout the study with active consideration of the potential for researcher bias to influence study design and analysis [17].

Integration occurred by comparison of the results obtained from separate qualitative and quantitative data collection and analysis. This approach enabled triangulation of the findings and took place during interpretation and development of the discussion [13].

## Results

Eighteen GPs participated in the study, 10 from the provincial city area and 8 from the metropolitan city area. The duration of the structured reflections ranged from 17 to 92 min (average 49 min) and semi-structured interview duration ranged from 25 to 99 min (average 49 min). A good degree of variation in practitioner experience, practice size and setting was attained within the sample (Table 1). The number of years participants had worked in general practice ranged from 3 to 48 years (average 19 years). The number of GPs in the practice, an indicator of practice size, ranged from 1 to 12 (average 6). Three participants worked in practices situated in a rural location.

**Table 1:** Participant demographics

*Participant # / Location*	*Gender*	*Years in general practice*	*# GPs in the practice*
01—Metropolitan city	F	26	7
02—Metropolitan city	M	3	8
03 – Provincial city	M	36	2
04—Provincial city	F	3	8
05—Provincial city—rural	M	48	2
06—Provincial city	F	5	10
07—Metropolitan city	F	11	7
08—Metropolitan city—rural	M	18	3
09—Provincial city	M	8	12
10—Provincial city	F	3	3
11—Provincial city—rural	M	35	2
12—Provincial city	M	19	1
13—Provincial city	F	36	4
14—Provincial city	M	31	3
15—Metropolitan city	M	25	3
16—Metropolitan city	M	5	10
17—Metropolitan city	F	6	6
18—Metropolitan city	F	21	3

*Key*: *SR* Structured reflection, *GP* General practitioner

### Frequency of medicines information needs

In the structured reflections, the eighteen participants reflected on a total of 642 consultations. The overall proportion of consultations where at least one medicines information need arose was 11% (n = 73/642) and the range for individual GPs was 0–28% of consultations.

### Nature of medicines information needs

**Fig. 1:**
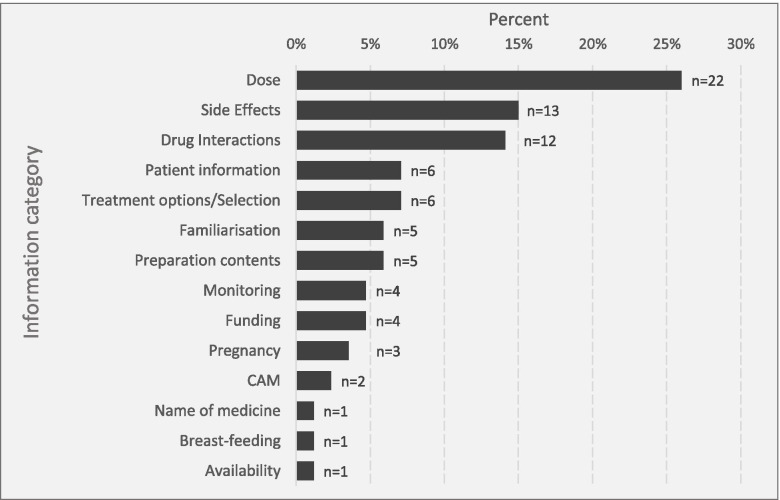
Structured reflection medicines information needs by category (n = 85)*. *In some cases, there was more than one information need per consultation. Familiarisation = review monograph to become familiar with medicine; CAM = Complementary and Alternative Medicines

Data derived from both quantitative (structured reflection) and qualitative (semi-structured interview) sources provided insight into the nature of general practice medicines information needs. Quantitative data gathered via structured reflection found a broad range of information needs with 14 different categories identified (Fig. 1). Dosing information was the most frequent, accounting for about a quarter (26%) of all reported needs. Side effect and drug interaction information were also prominent needs, accounting for 15% and 14% respectively.

**Fig. 2:**
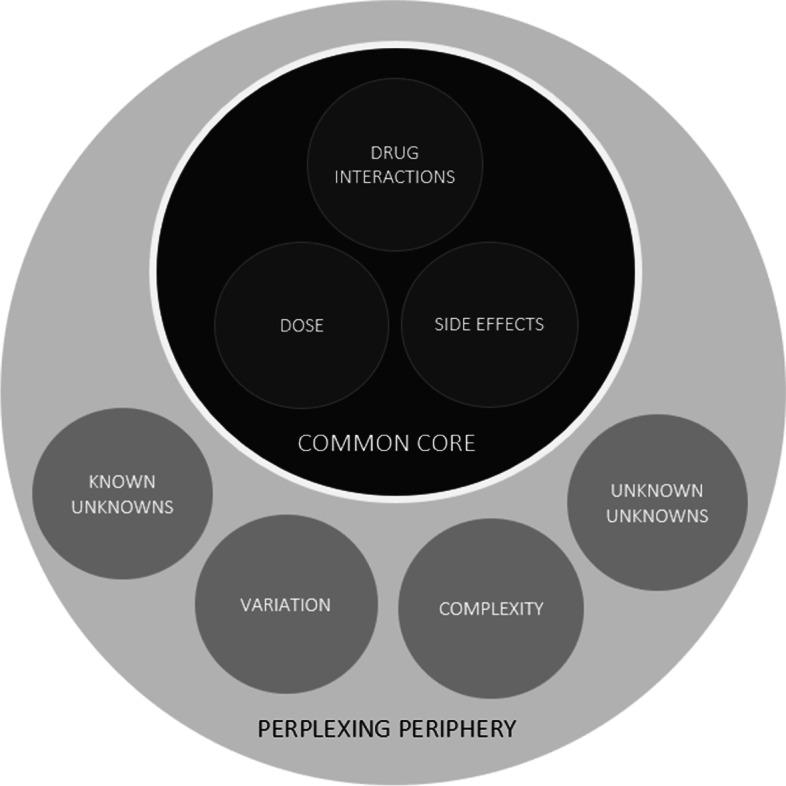
Thematic framework for the nature of general practice medicines information needs

Figure 2 provides an overview of the thematic framework resulting from analysis of the qualitative semi-structured interview data. Two key themes were identified by the research team as describing the nature of GP medicines information needs: (1) a common core and (2) a perplexing periphery. Consistent with the quantitative findings presented in Fig. 1, the three foci of medicines information needs in the ‘common core’ were dose, side effects and drug interactions. The term ‘perplexing periphery’ is used to encapsulate the less frequent but wide-ranging, unpredictable, and potentially complicated medicines information needs described by participants. Four sub-themes within the ‘perplexing periphery’ were identified and are outlined below with illustrative quotes.

#### Variation

Participants indicated that their medicines information needs were difficult to describe succinctly because of the large degree of variation.

*“It's the variety you need […] On one patient, you'll want a huge amount of depth, and the next patient it'll be one specific little question. […] the overall volume that you need is enormous […] I think it's a really difficult area and trying to make it more specific is quite hard." ****GP 08, Metro City***

"It's on-going […] you think you know it, and next week it's something new. It never, never stays the same […] It is literally always changing." **GP 01, Metro City**

#### Complexity

The complexity sub-theme encompasses issues of polypharmacy, multi-morbidity and specialist medicines described by participants.

*“Complex patients with […] multi-system disorders who are on an extensive list of medications, and you want to add one more and you want to gauge the probable impact of that on their kidney function, or interactions with what they’re already on.” ****GP 03, Provincial City***

*“Probably the most difficult is when people are attending specialists and they’re put on very sophisticated medicines, and they have to spend the rest of their lives with the stuff […] and they have other things wrong with them.” ****GP 05, Provincial City***

#### Known unknowns

‘Known unknowns’ captures a range of situations described by participants, such as where patients did not match criteria in guidelines or where guidelines were not available.

*“Where there aren’t hard and fast guidelines. For example, treating depression in pregnancy […] how to advise patients about risk around using their anti-depressant.” ****GP 10, Provincial City***

*“There are some specific areas where there’s not much information around, like the HRT [hormone replacement therapy] stuff […] I sort of Googled, and it’s either out of date, or the information I was looking for just wasn’t there…” ****GP 18, Metro City***

#### Unknown unknowns

Several participants highlighted the ultimate conundrum of not knowing what you do not know.

*“My big issue really comes back to knowing what I don’t know, and how you find these things out. Like I can read the PHARMAC*^3^* pamphlets on ‘do this, don’t do that’ but I often forget it straight away and forget that I’ve read it.” ****GP 06, Provincial City***

*“Information sharing is good, but probably we should check everything we do in an ideal world, but we can’t. And sometimes you don’t know what you don’t know, do you?” ****GP 01, Metro City***

### GP strategies for meeting their medicines information needs

Quantitative data from the structured reflections indicated that New Zealand-based core resources accessed electronically were used first line (MIMS^4^ 58% (n = 42/73), the New Zealand Formulary^5^ 30% (n = 22/73) and the Best Practice Advocacy Centre^6^ website 14% (n = 10/73). Google was the next most frequently used resource, featuring in 10% (n = 7/73) of the searches undertaken. In the semi-structured interviews, half of the participants included Google in their description of the strategy they use for meeting medicines information needs in practice.

### Factors influencing GP strategies for meeting medicines information needs

**Fig. 3:**
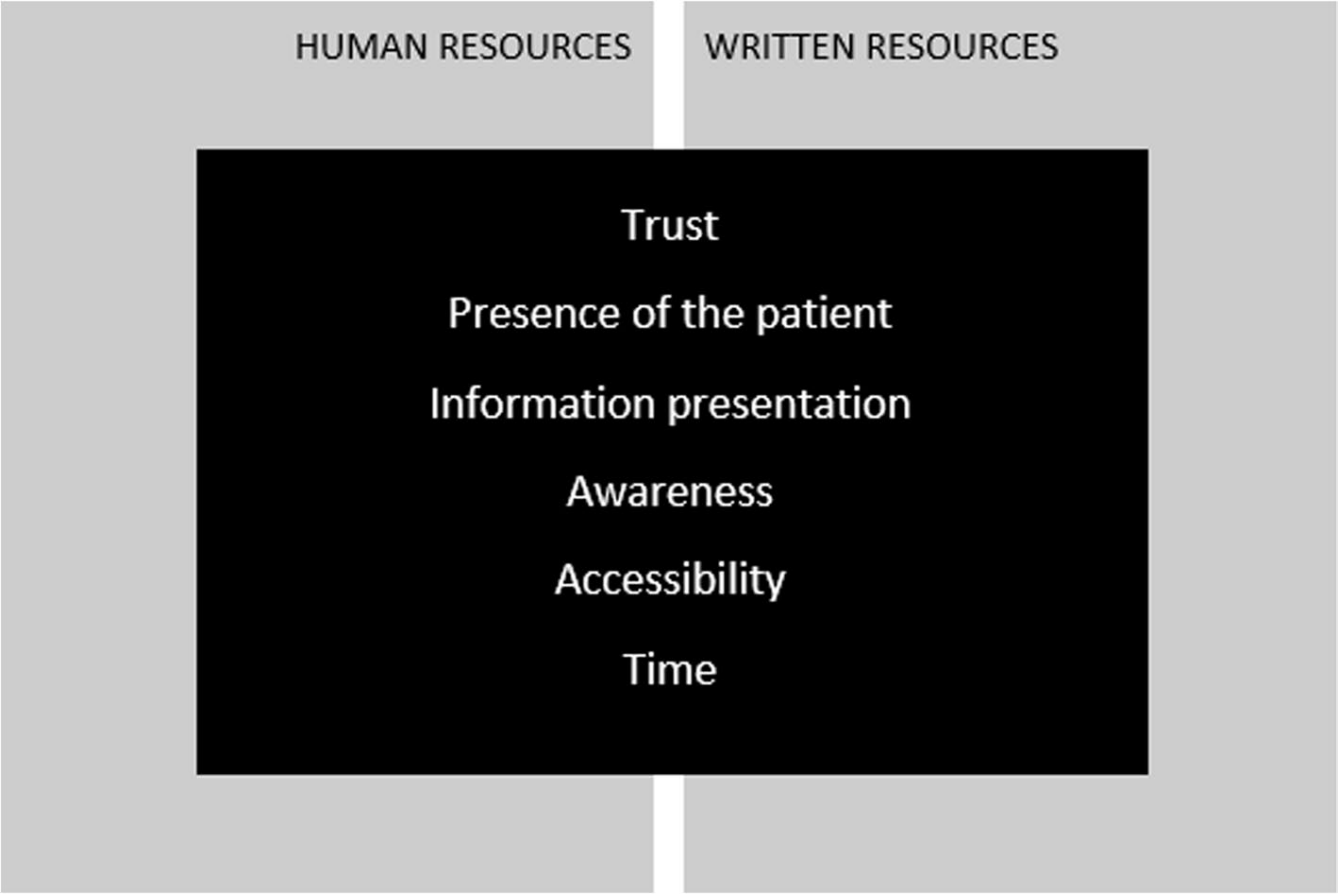
Factors influencing use of medicines information resources in general practice

Thematic analysis of the semi-structured interview data identified six overarching themes with potential to influence strategies used by GPs to meet medicines information needs (Fig. 3). The themes applied to written resources (both hard copy and online) and to human resources such as medical specialists, pharmaceutical company representatives and pharmacists.

#### Trust

Trust in the resource was a key influence on the use of both written and human resources:

*“If it’s appropriate sometimes I will use Google and just make sure that I come up with a reputable site. As long as I know what I’m doing really, it’s the same as looking at an old-fashioned textbook.” ****GP 09, Provincial City***

*“I used to work as a GP liaison at the hospital. I worked with her [a hospital medicines information pharmacist] on certain things, so there’s a woman there that I trust and know, so that makes it quite easy.” ****GP 13, Provincial City***

All participants commented on a strong relationship with local pharmacists especially around practicalities of medicines use such as cost, supply, and availability.

#### Presence of the patient

Participants indicated that the presence of the patient may affect their information seeking behaviour, in particular the potential for it to impact on patient trust in the doctor.

*“In fact, sometimes the easiest thing would be to Google it. But I don’t think that goes down well with patients […] I don’t think it’s a very good thing to do. And patients complain when other doctors have done that.” ****GP 08, Metro City***

*“Not everyone, but quite a lot of people object to the GPs looking stuff up. They’re sort of like; well you’re supposed to know everything. It’s much safer for you if I actually check stuff. It’s getting more acceptable […]” ****GP 17, Metro City***

#### Information presentation

Participants recognised the impact of information presentation on resource usability. This included both how information was presented and what information was presented:

*"The [commercial point-of-care medicines information resource integrated with prescribing software] on the computer is just a wall of text basically. Just lines of text with very little spacing, and the headings aren't in bold for different indications." ****GP 16, Metro City***

*"When you type up a prescription on [the patient management system] it has all these lists of interactions and a lot are baloney. And that's annoying, they cry wolf. We really only want the serious ones popping up as an alert […] So now I pretty much ignore it.” ****GP 01, Metro City***

A particular issue raised was the difficulty of searching electronic resources when you cannot recall a medicine name, coined ‘nominal aphasia’ by one participant.

*"I couldn't remember the name so it was hopeless looking at the Formulary […] I eventually had to go and scratch around and find my NZ Doctor magazine that advertised it…" ****GP 11, Provincial City***

#### Awareness

Awareness encompassed both knowing a resource existed and knowing how to use it optimally. There was varying awareness of different resources among participants.

*“For more detailed questions I wouldn’t ask my [local] pharmacist, I’d ask [hospital medicines information pharmacist name]. […] I was a house surgeon. We came across her then […] I suppose if you didn’t train in [location], I don’t know how you would know about her.” ****GP 06, Provincial City***

#### Accessibility

Ready accessibility within usual workflow patterns was also important for resource use:

“If it’s more than three or four clicks to get through to the place you want to get to, and then you’ve got to do your searching, then people just don’t do it.” **GP 15, Metro City**

“I might try asking my local pharmacist […] but I think they’re busy running a business.” **GP 06, Provincial City**

Participants reported that they did not use smart phones for information retrieval as their desk-top computers were more accessible and already in use for other tasks.

#### Time

Time constraints were commonly mentioned as a factor affecting information seeking:

*"The problem there is the question of time. You don't really, in our system where it allows you 15 minutes mostly for each patient, you've got to be very careful in your time management. It's a matter of remembering to do it later…" ****GP 05, Provincial City***

## Discussion

This study provides contemporary insight into the needs and challenges that GPs have in finding information to support optimal medicines use. It confirms they have regular needs with their most common searches being about dose, interactions and side effects [10, 18, 19]. It also suggests that outside this common core of medicines information needs, GPs are challenged by variation, complexity, ‘known unknowns’ and ‘unknown unknowns’. A deeper understanding of these most challenging elements of medicines use in general practice would enable information resources and support services to be tailored to better meet GP needs. A focus on gathering details of the ‘known unknowns’ encountered by GPs would allow assessment of whether they are truly unknown or whether increased searching time, different resources or different search strategies might help to reduce their occurrence. Knowledge gaps identified could then inform future research and strategies to support optimisation of medicines use [20, 21].

The increasing challenge of complexity associated with multi-morbidity and polypharmacy contributed to time being a key issue for participants in this study [22, 23]. When facing complex situations, the time and ability to locate, appraise, and interpret information about medicines to translate study findings into clinically meaningful information applicable to a specific patient is key. Medicines information services designed to provide timely patient-centred evidence-based clinical decision support are a potential option to help [24, 25]. Indeed, in the present study, several participants in one area still used the hospital-based medicines information service they were aware of from previously working in the local hospital. As well as addressing the time constraints of general practitioners, medicines information services provide a human connection [24]. This element may be important because a 2015 study of trainee GPs in Australia found that despite having trained in the ‘internet era’, human information sources were preferentially sought for more complex problems [26]. Other research supports the existence of a preference for expert human resources in more complex scenarios [27].

Although GPs in this study were positive about their relationship with local community pharmacists, they did not necessarily perceive them to be an accessible resource. There was limited awareness amongst participants of the support roles that pharmacists in different locations may play despite increasing literature indicating that general practice-based pharmacists can help to manage the growing burden of multi-morbidity and potential for inappropriate polypharmacy [28–30]. This suggests room for improved inter-professional collaboration to address medicines information needs in primary care and improve the safe and effective use of medicines.

Time limitations affected resource use in various ways in this study. For example, there was no evidence of traditional primary literature-searching using databases such as Medline which are known to be time-consuming [31]. Searching often transitioned from the core New Zealand-based medicines information references directly to Google, which is perceived as a quick route to wide-ranging information [32].

The predominant use of New Zealand-based references identified in the current work aligns with a study on physicians use of resources for evidence-based medicine across three countries where a preference for tools and publications produced within their own country was observed [33]. The authors of the previous study termed this a ‘cultural bias’, but for medicines information there are also practical trust-based drivers. Accurate local information is required regarding medicine availability, funding and regulatory status. Whether a medicine is approved by the local medicines regulatory authority may affect practitioner liability as well as the level of informed consent required for the patient.

This study identified that Google usage for medicines information needs occurs in general practice. The use of Google is not frequently mentioned in the wider literature but is consistent with one Australian study on internet use by GPs [34]. It could be more common in primary care because subscriptions for specialised medical information resources routinely available in secondary care are less affordable for smaller organisations. The ready accessibility and ease of use are also likely to be key factors [35, 36]. Google use warrants further examination given previous research suggesting that clinicians may be willing to accept lower quality or potentially biased information if it is available more quickly [6, 32].

Trust was identified as a factor affecting use of resources for medicines information in the present study. In line with the work of Sim et al*.*, participants in this study often loosely described consideration of the trustworthiness of information retrieved from the internet [34]. However, concerns have been raised in the literature about lack of systematic validation and the potential for hidden conflicts of interest with information sourced from the internet [37, 38]. A study of emergency medicine residents reported less accurate answers to clinical questions when using internet information located via Google [38]. Despite this, the residents were more confident in their answers when using Google. In a study of the online information searching of hospital physicians, Mikalef et al. also raised concerns about the use of non-authoritative information sources [35]. A better understanding of how health professionals are using Google to meet their medicines information needs is needed.

Another finding of this study was the presence of the patient as a factor affecting GP medicines information-seeking strategies. Participants felt that patients generally accepted that doctors would need to search for information at times. However, there was an overall sensitivity to patient perception of in-consultation searching activities. Though this issue has not been widely explored, one early investigation of physician information-seeking behaviour suggested that clinician information needs may be suppressed due to embarrassment [39]. A more recent study also found that some physicians preferred not to search for information during a consultation due to concerns that it would convey uncertainty or lack of knowledge [36]. However, a 2011 investigation of perceptions of in-consultation information-seeking found that most patients do not lose confidence in their physicians as a result. It was also suggested that physicians, especially those early in their career, tend to overestimate the potential for loss of patient confidence [40].

Our findings suggest the use of Google by doctors on the doctor-patient relationship warrants further attention. Google was perceived by participants as a useful information tool, but they were generally wary that it would be considered unprofessional by patients. Several mentioned that patients had commented to them with disapproval about other doctor’s use of Google. There is minimal research in this area though it has been suggested that internet search engine use by doctors may result in decreased patient confidence and a perception of reduced quality of care [40]. The need for a similarly accessible and usable portal but limited to quality medical information has been raised in the literature [35, 41].

The finding of significant drug interaction alert fatigue is consistent with international reports that alerts with low clinical relevance cause considerable clinician frustration and dissatisfaction [42]. Given the context of unprecedented, growing levels of polypharmacy [43] and the findings of this study that drug interactions are among the most common information needs expressed, this is of major concern and needs coordinated action by policy makers and software vendors.

The most frequently used resources in this study were accessed electronically via a desktop computer. This reflects the ease of access for GPs whose consultation workflow revolves around their computer, where they also document patient notes and generate prescriptions. In contrast with older studies [44], recent literature is increasingly reporting electronic resources as dominant tools in the information-seeking of medical students and qualified doctors including GPs [36, 45]. Despite the preference for electronic resources observed, smart phones were not used at all for medicines information retrieval by participants in this study. This is likely because there is no accessibility advantage to smart phones for GPs because their workflow revolves around a desktop computer. This suggests that when developing tools for GPs, the focus should remain on desktop applications rather than smart phone technology.

The mixed method approach used in this study provides a depth of understanding of the medicines information needs and challenges in general practice that could not be attained by either quantitative or qualitative findings alone. While some aspects of the nature of medicines information needs will vary with delivery of GP care in different countries, the factors influencing medicines information resource use identified in this study are likely to be relevant to GPs’ clinical practice in Western English-speaking countries with similar health care systems. Aspects contributing to the trustworthiness of the qualitative findings included diversity within the sample, participants reviewing their interview transcript to confirm accuracy, and attention to views that contradicted those of the majority during analysis.

A potential limitation for this study is the risk of sample bias. It is possible that GPs agreeing to take part had an interest in medicines information that was in some way different to those not taking part. There is also a risk of memory recall bias with self-report of information needs [6, 8]. Attempts were made to mitigate this effect by provision of a documentation booklet and only asking participants to reflect on consultations within the previous two days. Another limitation is that the quantitative data is primarily descriptive.

## Conclusion

General practitioners face challenges in meeting wide-ranging medicines information needs in patients with increasingly complex care needs. Understanding these challenges and considering the factors that influence resource use in practice provides important opportunity to optimise the design of written medicines information resources to better meet GP needs. This could contribute to more effective use of medicines and to a reduction of medication-related harm in primary care. The less frequent, unpredictable and complicated medicines information needs may be best addressed by individually responsive approaches such as collaboration with pharmacists and specialist medicines information support services.

## Supplementary Information

Additional file 1: Structure Reflection Data Collection Template.

Additional file 2: Structured Reflection Documentation Booklet.

Additional file 3: Semi-structured Interview Schedule.

## Data Availability

The datasets generated and analysed during the current study are available from the corresponding author upon reasonable request.

## Footnotes

1 Family practice physicians.

2 Ambulatory care.

3 *PHARMAC is a government agency that decides which medicines are publicly funded in New Zealand. Their role includes encouraging quality use of medicines.*

4 *MIMS is a commercial point-of-care medicines information resource.*

5 *New Zealand Formulary is a government funded online point-of-care medicines information resource.*

6 *Best Practice Advocacy Centre is an independent, not-for-profit organisation that delivers educational content to medical practitioners and other health professionals.*
