# Supplementary material for: A mixed methods study on medicines information needs and challenges in New Zealand general practice
Source: BMC Fam Pract. 2021 Jul 10;22:150. doi: 10.1186/s12875-021-01451-7 (PMC8272906; doi:10.1186/s12875-021-01451-7)

**Title**

**A mixed methods study on medicines information needs and challenges in New Zealand general practice**

**Authors**

Chloë Campbell^1,2,3^, Rhiannon Braund^1,4^, Caroline Morris^2^

^1^ School of Pharmacy, University of Otago, Dunedin, New Zealand

^2^ Department of Primary Health Care and General Practice, University of Otago, Wellington, New Zealand

^3^ Pharmaceutical Society of New Zealand, Wellington, New Zealand

^4^ New Zealand Pharmacovigilance Centre, University of Otago, Dunedin, New Zealand

**Corresponding author**

Chloë Campbell

chloecampbell@mail.com

***Additional file 1: Structured Reflection Data Collection Template***


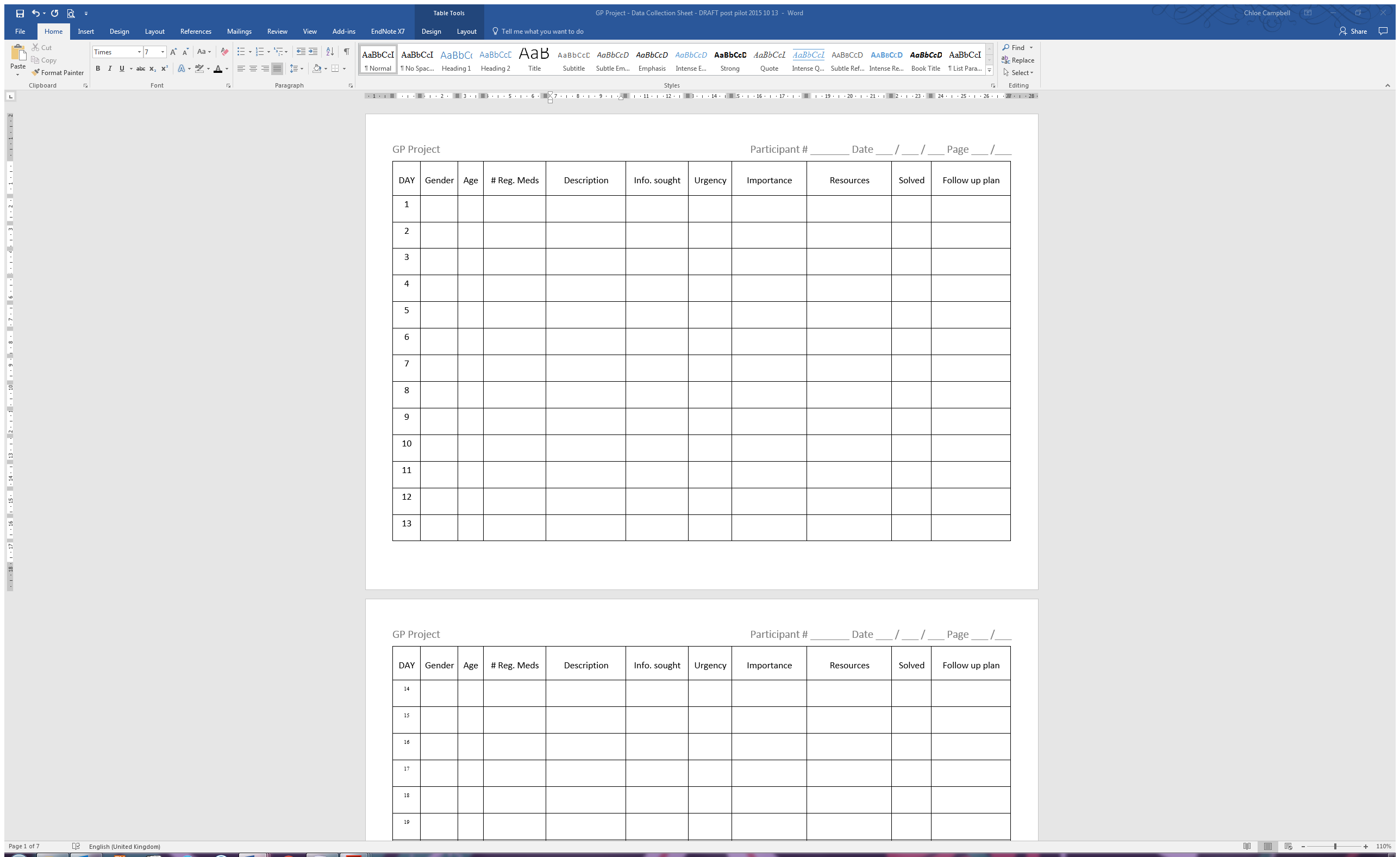

Supplement: Supplementary file 1 — Structure Reflection Data Collection Template. [file 12875_2021_1451_MOESM1_ESM.docx]
