# Supplementary material for: A mixed methods study on medicines information needs and challenges in New Zealand general practice
Source: BMC Fam Pract. 2021 Jul 10;22:150. doi: 10.1186/s12875-021-01451-7 (PMC8272906; doi:10.1186/s12875-021-01451-7)
Supplement: Supplementary file 2 — Structured Reflection Documentation Booklet. [file 12875_2021_1451_MOESM2_ESM.docx]

**Title**

**A mixed methods study on medicines information needs and challenges in New Zealand general practice**

**Authors**

Chloë Campbell^1,2,3^, Rhiannon Braund^1,4^, Caroline Morris^2^

^1^ School of Pharmacy, University of Otago, Dunedin, New Zealand

^2^ Department of Primary Health Care and General Practice, University of Otago, Wellington, New Zealand

^3^ Pharmaceutical Society of New Zealand, Wellington, New Zealand

^4^ New Zealand Pharmacovigilance Centre, University of Otago, Dunedin, New Zealand

**Corresponding author**

Chloë Campbell

chloecampbell@mail.com

***Additional file 2: Structured Reflection Documentation Booklet***

***Introduction to documentation booklet***

This booklet is to assist your recall during the *structured reflection* - please feel free to use it to make notes about any medicines-related issues or information needs that arise for you during the two days prior to the scheduled session. If needed to aid recall, please use initials rather than names in your notes to preserve patient confidentiality.

During the structured reflection itself, the researcher will ask a consistent sequence of questions about each consultation to gather information about medicines-related issues or information needs. The session will be digitally audio-recorded.

Please do not hesitate to contact me if you have any questions about the project - mobile # or e-mail*.*

***The booklet contained space for multiple medicines information needs to be documented with the following headings:***

Medicines-related issue, question or information need

Resources used?

Outcome e.g. information found/problem solved or more follow up required
