# Supplementary material for: A mixed methods study on medicines information needs and challenges in New Zealand general practice
Source: BMC Fam Pract. 2021 Jul 10;22:150. doi: 10.1186/s12875-021-01451-7 (PMC8272906; doi:10.1186/s12875-021-01451-7)
Supplement: Supplementary file 3 — Semi-structured Interview Schedule. [file 12875_2021_1451_MOESM3_ESM.docx]

**Title**

**A mixed methods study on medicines information needs and challenges in New Zealand general practice**

**Authors**

Chloë Campbell^1,2,3^, Rhiannon Braund^1,4^, Caroline Morris^2^

^1^ School of Pharmacy, University of Otago, Dunedin, New Zealand

^2^ Department of Primary Health Care and General Practice, University of Otago, Wellington, New Zealand

^3^ Pharmaceutical Society of New Zealand, Wellington, New Zealand

^4^ New Zealand Pharmacovigilance Centre, University of Otago, Dunedin, New Zealand

**Corresponding author**

Chloë Campbell

chloecampbell@mail.com

***Additional file 3: Semi-structured Interview Schedule***

The main headings indicate the key areas to be covered. The questions listed are examples and the interviewer may adjust questioning at the time of the interview as appropriate. Probes may also be used to delve further as needed.

INTRO DEMOGRAPHICS

- How many years have you been working in general practice?
- Have you ever worked in general practice outside New Zealand? If so, where?
- Do you have an area or areas of ‘special interest’? If so, what are they?
- What training around information literacy have you had? Undergrad/Post grad/CE

MEDICINES INFORMATION NEEDS AT POINT OF CARE

- How would you describe the nature of your information needs about medicines? What do you feel are the commonest type of questions/dilemmas you seek extra information to solve?
- How would you describe the strategy you usually use for seeking information about the use of medicines or solving problems related to medicines? How do you decide which strategy to use?
- What kind of questions are the hardest to find answers to?
- Do you tend to look things up in the consultation or after?
- How would you describe your access to information about medicines at the point of care?
- What are your views on the format of medicines information resources currently available?
- What do you consider to be the most useful resources about medicines?
- How do you assess the trustworthiness of [internet] information sources?
- How do you make contact with specialists or colleagues…?
- What are your thoughts on Google or the internet in general as a medicines information resource?

BARRIERS TO MEETING MEDICINES INFORMATION NEEDS IN PRACTICE

- What would you say are the barriers that prevent you from meeting information needs about medicines in practice?
- How do you feel about searching for information when the patient is with you?
- Some people I have talked to have mentioned feeling a bit shy in relation to information needs, i.e. the feeling of I should probably know this so I can’t ask… what are your thoughts on this?

SOLUTIONS TO MEETING MEDICINES INFORMATION NEEDS IN PRACTICE

- What are potential solutions that might help you better access the information and support you need around the use of medicines?
- How could medicines information resources be made more user-friendly to GPs?
- Can you describe your ideal situation in terms of information, advice and support around the use of medicines?

STAYING UP TO DATE WITH MEDICINES INFORMATION RESOURCES

- How do you find out about new medicines?
- How do you find out about (new) medicines information resources?
- Do you subscribe to any e-mail updates about medicines?
- Do you use social media for professional purposes?
- Do you use smart phone for professional information retrieval? Any particular apps?

PHARMACIST ROLE IN MEDICINES INFORMATION – CURRENT & POTENTIAL

- How do you see the role of pharmacists in helping you to solve medicines-related questions/ issues?
- How close is the nearest community pharmacy? What is your working relationship like? Do you ask them medicines-related questions? What type of questions?
- Do you work with a PHO pharmacist? Do you ask them medicines-related questions?
- Are you aware of hospital-based medicines information services? Have you used or would you use a hospital-based medicines information service? How often might you use it?
- Would you have a preference between local community pharmacist / PHO pharmacist / hospital pharmacy-based medicines information service?
- When would you consider asking a pharmacist? What type of question would you be more likely to ask a pharmacist?

*Any other thoughts in general about medicines information needs?*

EXIT DEMOGRAPHICS

| Male / Female |
| --- |
| Number of GPs in practice? |
| List size - # patients served? Practice and individual GP |
| Year of registration?  Trained in NZ? Other?  Vocationally registered?  Current FTE or sessions per week? |
| Medicines Information References in GP’s office?  Hard copy – books  Hard copy – journals  Electronic – books  Electronic – journals  Electronic – websites  Electronic - databases  Electronic - apps |
| Other Medicines Information References available within practice? |
| Practice budget for medicines-related resources? |
